# The Physical Interaction of Myoblasts with the Microenvironment during Remodeling of the Cytoarchitecture

**DOI:** 10.1371/journal.pone.0045329

**Published:** 2012-09-18

**Authors:** Daniel J. Modulevsky, Dominique Tremblay, Corinne Gullekson, Nickolay V. Bukoresthliev, Andrew E. Pelling

**Affiliations:** 1 Department of Physics, Centre for Interdisciplinary NanoPhysics, University of Ottawa, Ottawa, Ontario, Canada; 2 Department of Biology, University of Ottawa, Ottawa, Ontario, Canada; 3 Institute for Science Society and Policy, University of Ottawa, Ottawa, Ontario, Canada; Dalhousie University, Canada

## Abstract

Integrins, focal adhesions, the cytoskeleton and the extracellular matrix, form a structural continuum between the external and internal environment of the cell and mediate the pathways associated with cellular mechanosensitivity and mechanotransduction. This continuum is important for the onset of muscle tissue generation, as muscle precursor cells (myoblasts) require a mechanical stimulus to initiate myogenesis. The ability to sense a mechanical cue requires an intact cytoskeleton and strong physical contact and adhesion to the microenvironment. Importantly, myoblasts also undergo reorientation, alignment and large scale remodeling of the cytoskeleton when they experience mechanical stretch and compression in muscle tissue. It remains unclear if such dramatic changes in cell architecture also inhibit physical contact and adhesion with the tissue microenvironment that are clearly important to myoblast physiology. In this study, we employed interference reflection microscopy to examine changes in the close physical contact of myoblasts with a substrate during induced remodeling of the cytoarchitecture (de-stabilization of the actin and microtubule cytoskeleton and inhibition of acto-myosin contractility). Our results demonstrate that while each remodeling pathway caused distinct effects on myoblast morphology and sub-cellular structure, we only observed a ∼13% decrease in close physical contact with the substrate, regardless of the pathway inhibited. However, this decrease did not correlate well with changes in cell adhesion strength. On the other hand, there was a close correlation between cell adhesion and β1-integrin expression and the presence of cell-secreted fibronectin, but not with the presence of intact focal adhesions. In this study, we have shown that myoblasts are able to maintain a large degree of physical contact and adhesion to the microenvironment, even during shot periods (<60 min) of large scale remodeling and physiological stress, which is essential to their in-vivo functionality.

## Introduction

Many living cells proliferate and survive while being strongly associated with the extracellular matrix (ECM), which can have significant effects on their functions [1], [2]. The interaction of cells with the ECM and microenvironment is largely mediated through a complex set of interactions between trans-membrane and internal protein complexes and the cytoskeleton (CSK) [1], [2]. Trans-membrane protein complexes such as integrins are composed of α and β subunits that can assemble into twenty-four different heterodimers [3], [4]. These dimers bind to ECM proteins such as collagen and fibronectin and possess internal cytoplasmic domains that interact with focal adhesion (FA) proteins [1], [2]. FAs are complex structures that employ linker proteins such as vinculin, zyxin, talin and paxillin, in order to integrate the FA site with the actin filament network and the rest of the CSK [2], [5]–[7]. The CSK is a highly cross-linked network of major filament systems composed of actin, microtubules (MTs) and various intermediate filaments, which ultimately allow communication, the transmission of mechanical signals and transport of materials throughout the entire cell [1], [2]. FAs and integrins mediate the adhesion and interaction of cells with the underlying substrate and allow cells to sense mechanical cues (mechanosensitivity) and respond to local mechanical forces (mechanotransduction) arising in the microenvironment [2], [8]–[11]. Indeed, stretching of the FA and integrin protein complexes through acto-myosin contractility is believed to increase the interaction of FA proteins with actin filaments and lead to integrin clustering [12]. Myosin-II (myoII) is also required for the recruitment of focal adhesion kinase (FAK), zyxin and vinculin; it is not the case for the recruitment of paxillin, talin, and α1-integrin [13]. Conversely, the loss of acto-myosin contractility results in the disassembly of well-defined FA sites and integrin clustering which leads to a loss of cell adhesion [14]–[16].

Flow chamber assays have been extensively employed to study the fundamental nature of cell adhesion to the microenvironment to better understand the role that integrins and all FA proteins play in cell-substrate interaction. Previous studies have shown that adhesion strength is directly proportional to the number of active integrin bonds that link the cell with its substrate [16]–[19]. Although it has been acknowledged that integrins contribute the most to the strength of the adhesion, focal adhesion kinase (FAK) also contributes to adhesion strengthening by modulating the binding of integrin available on the cell membrane [18] and inducing a rapid increase in adhesion strength upon integrin activation [15], [18]. Several studies used microprinting techniques to create functionalized islands of defined size on which cells were firmly attached and then exposed to a shear stress in order to demonstrate a direct relationship between cell contact area and adhesion strength [12], [15], [16]. Traditionally, the area bounded by the cell contour has been taken as a measure of cell contact area (or cell size). However, as revealed by interference reflection microscopy (IRM) (sometimes referred to as or reflection interference contrast microscopy), many cells do not display homogenous contact with the substrate throughout their total spreading area [20]–[23]. These heterogeneously distributed regions of close cell-substrate association indicate that only a fraction of their total spreading area is in close physical contact with the surface. Although IRM was developed for the study of thin films, it has many applications has become useful for the study of cell-substrate interactions [14]–[16], [20]–[31]. Indeed, it is possible to characterize the nature of close cellular contact with an underlying substrate by examining dark regions in the IRM image [20]–[23]. By quantifying the total area of low intensity pixels one can define a close contact area (CCA), which corresponds to the total area of the cell membrane that is within ∼100 nm of the substrate [20]–[23]. This subtle, yet important, distinction between total cell spreading area (or size) and CCA has not been explored previously and it is unclear how CCA relates to the dynamics of cellular adhesion.

In this study, we employed IRM to study the nature of the interaction of C2C12 mouse myoblast cells with a substrate. Myoblasts are muscle pre-cursor cells that fuse and form myotubes (myogenesis). This process is highly mechanosensitive, requiring an intact CSK and strong contact and adhesion to the microenvironment [32]–[38]. It has been demonstrated that myoblasts cultured on hydrogels of increasing stiffness leads to optimal myogenesis on substrates having an elasticity similar to resting muscle tissue [37]. Conversely, the in-vivo microenvironment of a myoblast is highly dynamic, constantly undergoing mechanical stretch and compression. Importantly, it has been shown that when cells experience stretch and compression, dramatic CSK remodeling, depolymerization and fluidization takes place [39], [40]. Therefore, in the case of myoblasts, it is unclear if significant remodeling of the CSK and loss of acto-myosin contractility results in a significant loss in CCA and/or adhesion to a substrate.

The objectives of this study were to characterize how the induction of CSK remodeling and inhibition of acto-myosin contractility alters CCA, cell spreading area and adhesion. We demonstrate that myoblasts are capable of maintaining strong adhesion and CCA during CSK depolymerization, the loss of acto-myosin contractility and FA remodeling over short timescales (up to 1 hour). Evidence suggests that during these remodeling processes, integrins and cell-secreted fibronectin remain intact which act to maintain cell adhesion. As well, we also observed a complex relationship between CCA, cell spreading area and adhesion. It is important for myoblasts to maintain contact and adhesion with the microenvironment even as it undergoes mechanical or physiological stress, in order to maintain and rebuild muscle tissu e. It is clear from this study that myoblasts are able to maintain adhesion and CCA during dramatic remodeling of the cytoarchitecture over short time scales. We speculate, that this may be part of a coping mechanism to remain in contact with the microenvironment during short periods of activity and during the early myogenic processes [8], [37], [41].

## Results

### Effect of Inhibitors on the Morphology of the Cytoskeleton

**Figure 1:**
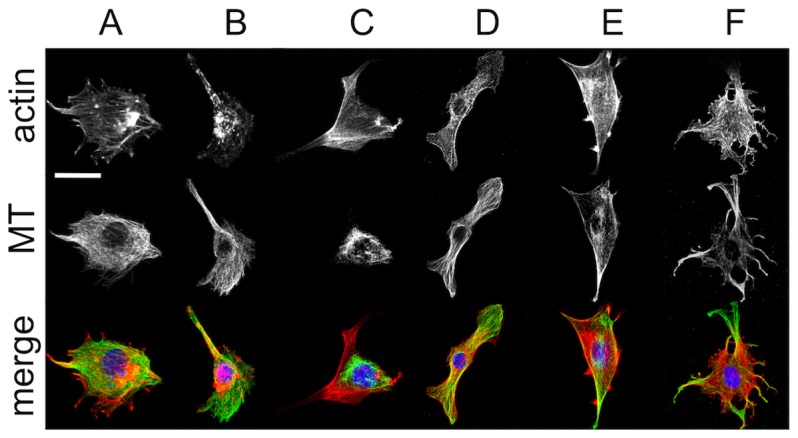
Fixed and stained images of the C2C12 myoblast cytoskeleton. Control cells are show in (**A**) and cells treated with anti-cytoskeletal inhibitors for 60 min are shown in (**B**) Cyt-D, (**C**) nocodazole, (**D**) ML-7, (**E**) Y-27632 and (**F**) blebbistatin. The actin and MT cytoskeleton are shown at the top and middle row respectively. A merged image of the actin (red), MT (green) and nucleus (blue) are in the bottom row. Scale bar = 20 µm and applies to all images.

C2C12 mouse myoblast cells were treated for one hour with one of the following inhibitors: the actin depolymerizing inhibitor cytochalasin-D (CytD), the microtubule (MT) depolymerizing inhibitor nocodazole, the rho kinase (ROCK) inhibitor (Y27632), the myosin light chain kinase (MLCK) inhibitor (ML7) and an inhibitor which directly inhibits myoII (blebbistatin). After treatment, cells were fixed and stained to visualize the actin CSK, the MT CSK and the nucleus with laser scanning confocal microscopy (LSCM) (Fig. 1). As can be seen in the figure, untreated cells display classic adherent cell actin stress fibres, MT and nuclear morphologies (Fig. 1A). Treatment with CytD results in complete loss of filamentous actin while the MT remains intact (Fig. 1B). A similar effect is observed in response to Y27632 (Fig. 1D). Conversely, nocodazole causes the loss of filamentous MTs while leaving actin stress fibres intact (Fig. 1C). MyoII inhibition with ML7 and blebbistatin caused the loss of actin stress fibres, while MTs remain intact (Fig. 1E, F).

### Time-lapse IRM Imaging and Analysis

**Figure 2:**
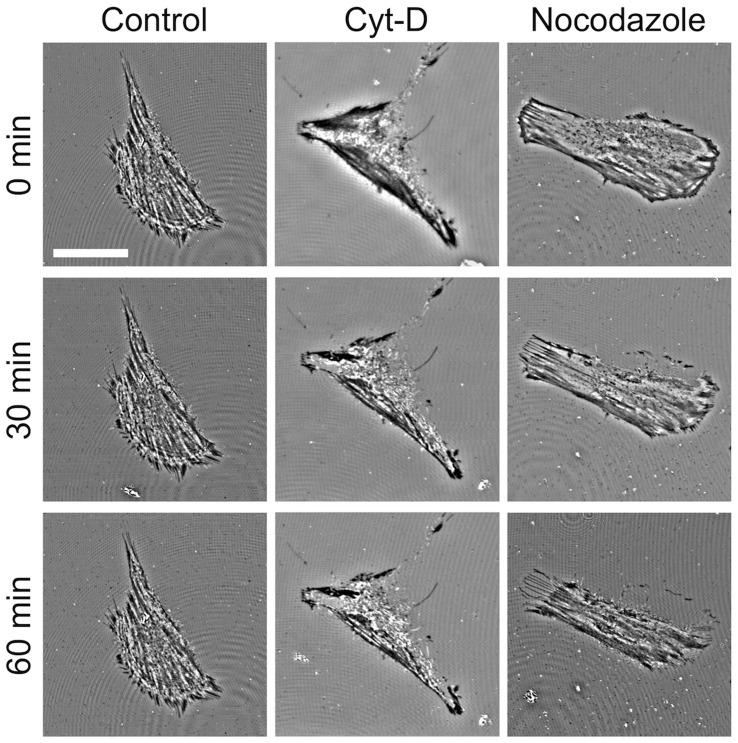
Time-lapse IRM imaging on inhibitor treated C2C12 cells. IRM time-lapse imaging on C2C12 cells before, during and after a 60 min exposure to Cyt-D and nocodazole in comparison to control cells (scale bar = 20 µm and applies to all images). Cells treated with Cyt-D and nocodazole display decrease in the dark regions of the cell over the course of 60 mins. The control cells exhibit no change in the low intensity regions of the cell. ML-7 and Y-27632 treatments (data not shown) displayed similar trends as those shown for the Cyt-D and nocodazole treatments. Scale bar = 20 µm and applies to all images.

In order to investigate the effects of inhibitor treatments on cellular contact with the substrate, we performed IRM time-lapse imaging on cells before and during a 60 min exposure to each inhibitor (Fig. 2). IRM images were acquired every 15 mins for a total of 60 mins without (control) or following the addition of an inhibitor (n = 3 cells for each condition). In every case the t = 0 min image is acquired immediately prior to the introduction of an inhibitor. Control cells imaged over 60 mins reveal almost no significant changes in the IRM images, aside from small intensity fluctuations. In the case of treatment with CytD, it can be observed that the low intensity regions of the cell at t = 0 min (corresponding to close contact between the cell and substrate) diminish over the course of 60 mins indicating a decrease in total CCA. Likewise, similar results were also observed for nocodazole and Y27632, ML7 and blebbistatin (data not shown).

**Figure 3:**
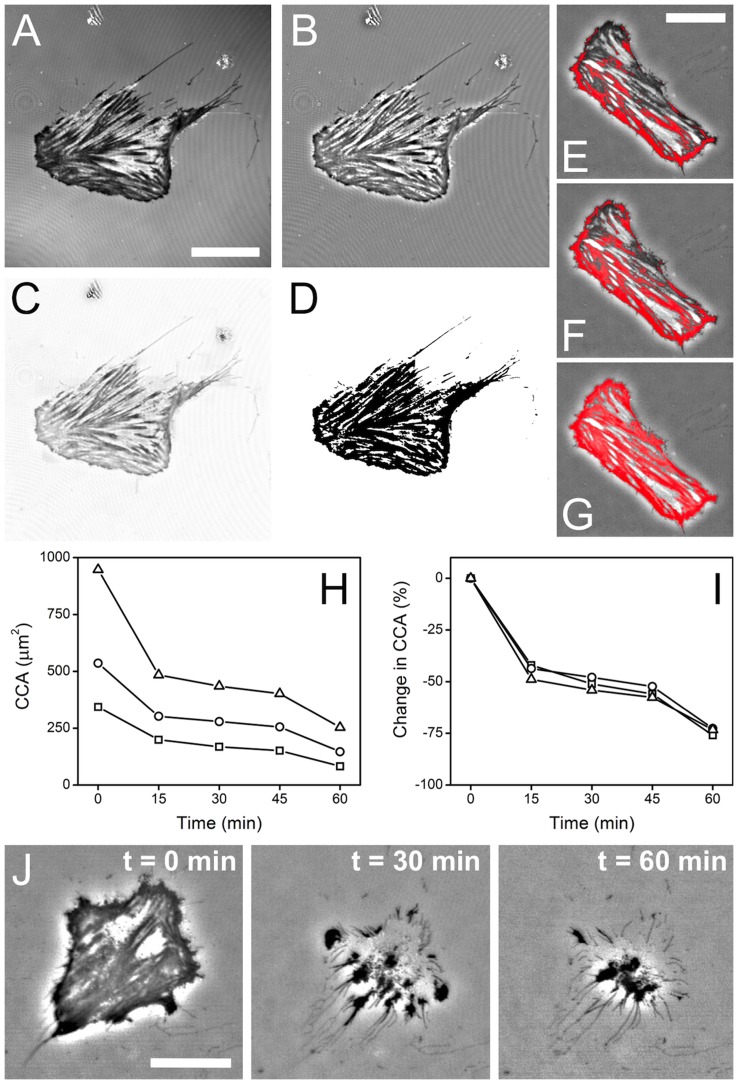
Computational analysis of cell contact area in IRM images. (**A**) Raw IRM image of a cell adhered to a glass substrate (scale bar = 25 µm, and applies to all). (**B**) The same image after applying a FFT band-pass filter to create a uniform intensity background. (**C**) The image after applying a background subtraction/lightening filter to isolate sites of close cellular contact from the background. (**D**) The image is then thresholded to form a binary image where regions of close contact (corresponding to grey values lower than the background intensity in (**C**)) are set to zero intensity. Artifacts arising from debris are manually removed and then the zero intensity pixels are summed to provide a measure of cell CCA. (**E–G**) The effect of different user specified thresholds (shown in red) yield different values of the (**H**) absolute CCA as a function of time. (**I**) Normalizing the data yields the change in CCA and all three curves fall on one another. (**J**) Time-lapse imaging of C2C12 cells during exposure to EDTA. All cells showed a consistent decrease in contact area of about 75% and that CCA is calcium dependent.

**Figure 4:**
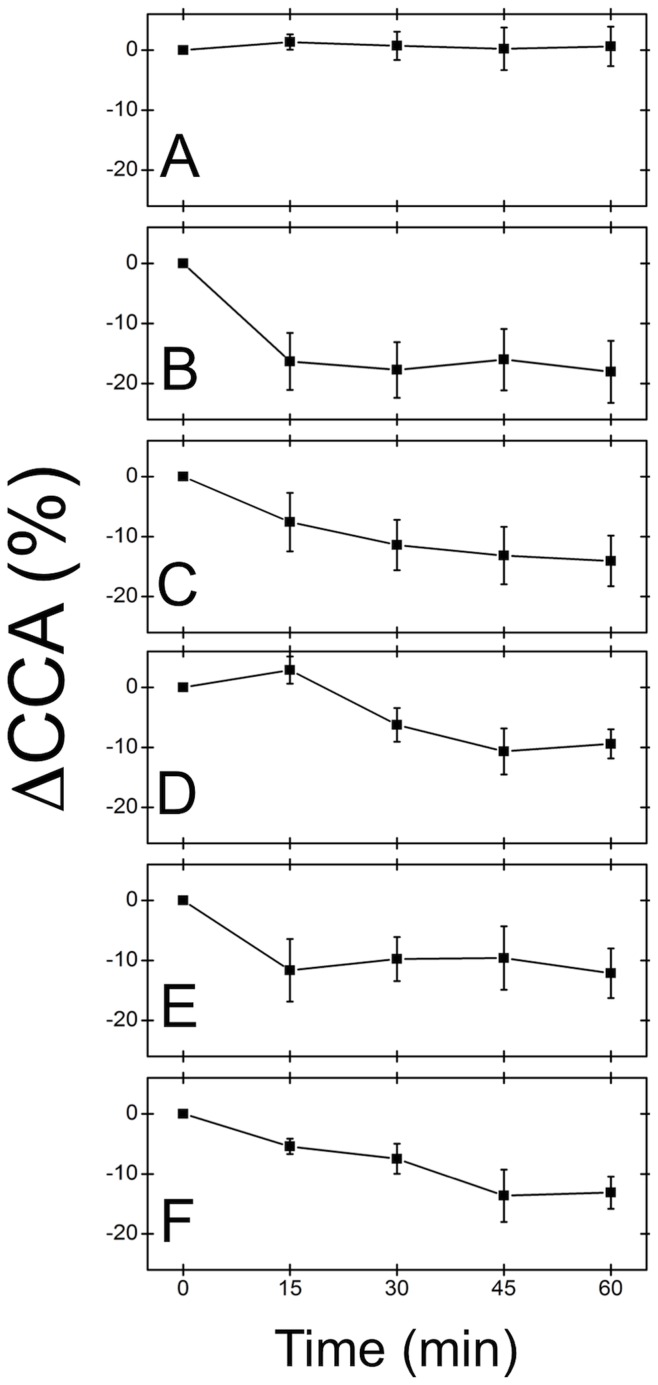
Dynamic changes in C2C12 close contact area over 60 mins. Cells were treated with various inhibitors and IRM images were acquired every 15 mins for 60 mins. (**A**) Control, (**B**) Cyt-D, (**C**) nocodazole, (**D**) Y-27632A, (**E**) ML-7 and (**F**) blebbistatin treated cells (n = 3 cells for each condition). In each case, except for the control, a significant decrease in contact area occurs after 60 mins of exposure to each inhibitor. However, there was no statistically significant dependence on the type of inhibitor used. On average, the ΔCCA was ∼13% for each condition, except for the control.

In order to quantify the CCA we wrote a script in ImageJ to extract the total area of pixels corresponding to close contact (Fig. 3) in a manner similar to those described previously [20]–[23]. Low to median intensity pixels in intensity normalized IRM images typically correspond to regions of close physical contact between the cell and substrate when acquired with a high NA objective at visible wavelengths. To calculate CCA, raw IRM images (Fig. 3A) were FFT band-pass filtered resulting in a uniform-intensity background due to the removal of low frequency shadowing typically observed in the raw images (Fig. 3B). In order to further isolate the cell from the substrate, the background was smoothed and lightened using the background subtraction plugin (Fig. 3C). The resulting image was then thresholded to form a binary image (Fig. 3D) where the regions of close contact are distinguished from the background (any artifacts caused by debris on the glass substrate were easily identified and were subtracted manually). The zero intensity pixels in the binary image were then simply summed to provide a measure of CCA. As can be seen in Fig. 3A and D, the image processing accurately captures regions of median to low intensity pixels in the raw IRM images while ignoring high intensity pixels and the background. In all cases, the filters and filter settings were kept constant for all images in a single time-lapse sequence and for all sets of data under varying inhibitor and control conditions. However, as these settings are user defined it is possible to introduce bias into the measurement. In Fig. 3E–G, three user defined thresholding settings are shown which capture varying amounts of the low to median intensity pixels in the IRM image. The cell was then treated with 10 µM EDTA for 60 mins and the absolute CCA as a function of time was calculated (Fig. 3H) using the thresholding settings shown (Fig. 3E–G). By normalizing the data to a percentage change in CCA we show that the three plots are very similar. Therefore, as long as the user sets a reasonable threshold (i.e. capturing only regions within the cell), the change in CCA can be compared between individual cells and experiments. This methodology allowed us to measure the change in CCA over time for control cells and cells exposed to one of the five inhibitors (Fig. 4). Importantly, cells treated with EDTA consistently demonstrated a ∼75% decrease in CCA (Fig. 3I, J). Indeed, cells were observed to come off the tissue culture plate (confirmed by focusing above the substrate after the EDTA treatment) and only leaving behind debris. This experiment confirms that dark areas in the IRM images were due to close association between the cell and substrate and that this interaction is dependent on the presence of calcium.

### Cell Size Dynamics in Response to Inhibitor Treatments

The average change in cell CCA, under a given set of conditions and as a function of time, was quantified as above and is shown in Fig. 4. Control cells (Fig. 4A) reveal no significant changes in CCA over the 60 min imaging time. However treatment with CytD (Fig. 4B) reveals a rapid decrease in CCA within 15 mins of treatment that remains fairly constant over the remaining imaging time. Nocodazole treatment (Fig. 4C) results in a slow decrease in CCA over 60 mins. Cells exposed to Y27632 (Fig. 4D) only begin to decrease CCA after 30 mins of exposure to the inhibitor. Cells treated with ML7 (Fig. 4E) respond in a similar manner as the CytD treated cells, with rapid decrease in CCA within 15 mins followed by little change. Finally, blebbistatin treatment also results in a slow decrease in CCA, similar to nocodazole treatment (Fig. 4F). What can be observed is that treatments always result in a statistically significant decrease in contact area when compared to the control (p<0.04 for each case). However, there is no clear statistical difference between any of the inhibitors after 60 mins (p>0.1 for each case). Overall, after a 60 min treatment with any inhibitor the average decrease in CCA was ∼13%. On the other hand, the absolute cell spreading area (determined by calculating the total area defined by the cell boundary) was shown to significantly decrease by ∼50% in response to the various inhibitors compared to the control (p<0.01) with no significance between them (p>0.1), except in the case of blebbistatin which does not change with respect to the control (Table 1). In order to confirm that the change in CCA was not simply due to the cells contracting and lifting off from the substrate, we measured cell height (Table 1) by recording the distance between the apical and basal membrane above and below the nucleus in LSCM images (n = 10 cells for each condition). We observed no appreciable change in cell height after expose to each inhibitor (p>0.2). It is tempting to interpret this as due to a decrease in volume, however, volume measurements with traditional confocal microscopy are challenging due to the nature of the axial PSF. Therefore, we only conclude the apparent decrease in CCA is not due to the cells moving up and away from the surface.

**Table 1:** CCA, Cell Height and Cell Area after 60 min of exposure to various inhibitors.

Treatment	Change in CCA (%)	Cell Height (µm)	Cell Area (µm^2^)
Control	1±3%	11.0±2.0	2519±815
CytD	−18±5%	10.2±1.2	1247±503
Nocodazole	−14±4%	11.0±1.2	1186±814
Y27632	−9±2%	10.1±1.3	1219±410
ML7	−12±4%	10.2±2.7	1376±659
Blebbistatin	−13±3%	9.6±1.8	2687±826

### Changes in Adhesion Strength during Inhibitor Treatments

**Figure 5:**
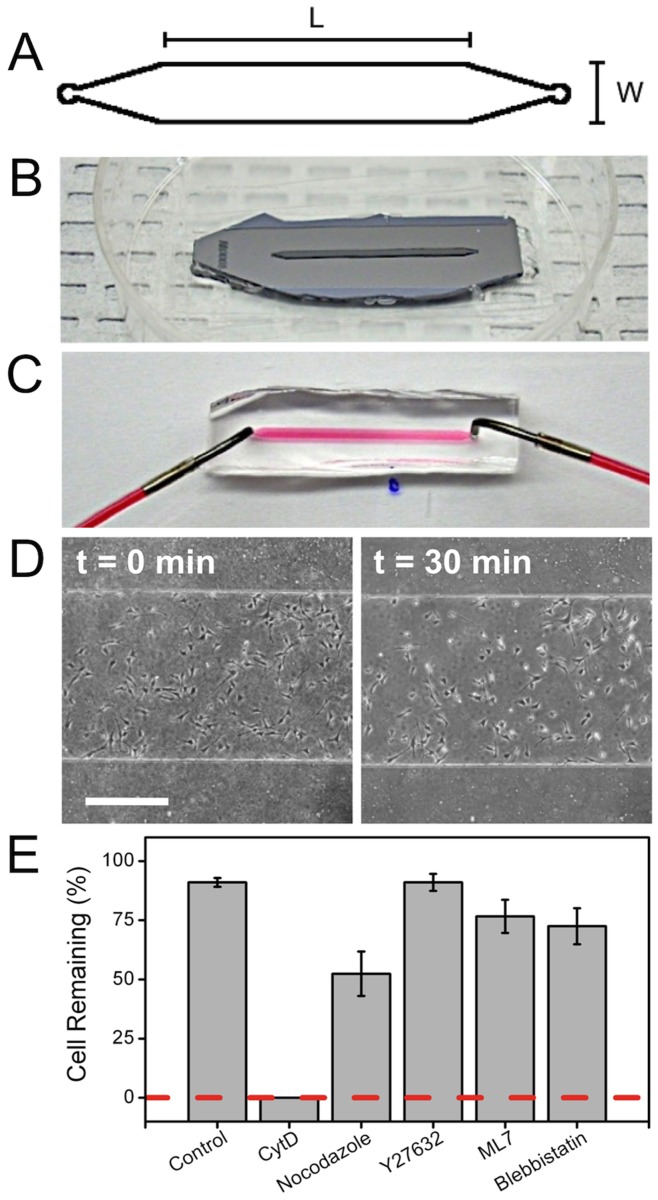
Flow chamber adhesion assay and changes in cell adhesion strength. (**A**) In face flow chamber dimensions: L = 2 cm and W = 1 mm. (**B**) SU-8 master used to fabricate flow chamber with a height of 160 µm. (**C**) Water with red dye shows the flow chamber working principle with bent stainless steel needle attached to the inlets and outlets allowing media to be pumped through the chamber. (**D**) Typical adhesion assay where cells were exposed to shear stress over a period of 30 min. Cells were counted before and after flow to quantify changes in cell-substrate adhesion strength (scale bar = 500 µm). (**E**) Bar graph showing the ratio of cells remaining on the glass substrate after exposition to a shear stress of 65 Dyne/cm^2^ over a period of 30 min. There is no significant difference between the control and Y27632 treatment (p>0.1). All other treatments yield a significant decrease relative to the control (p<0.04).

In order to examine if changes in CCA are correlated to changes in cell adhesion strength we fabricated a parallel plate flow chamber (Fig. 5A–C). Cells were pretreated with each inhibitor for 60 min prior to the onset of a high flow producing a shear stress of 65 Dyne/cm^2^ [42]. The percentage of cells remaining after 30 min of continuous shear stress allowed us to qualitatively determine the change in adhesion strength relative to untreated (control) cells (Fig 5E, D). Consistent with previous work, we observed a ∼9% decrease in the number of control cells exposed to shear stress [14]. What is immediately evident is that loss of the actin CSK in response to CytD treatment results in a complete loss of cell adhesion as no cells remained after 30 min of shear stress. We also observed a significant decrease in the number of cells remaining after treatment with nocodazole, ML7 and blebbistatin (p<0.05) when compared to control cells. Interestingly, ROCK inhibition with Y27632 did not cause a statistically significant decrease in remaining cells compared to the control (p>0.9).

### Remodeling of Adhesion Structures in Response to Inhibitor Treatments

**Figure 6:**
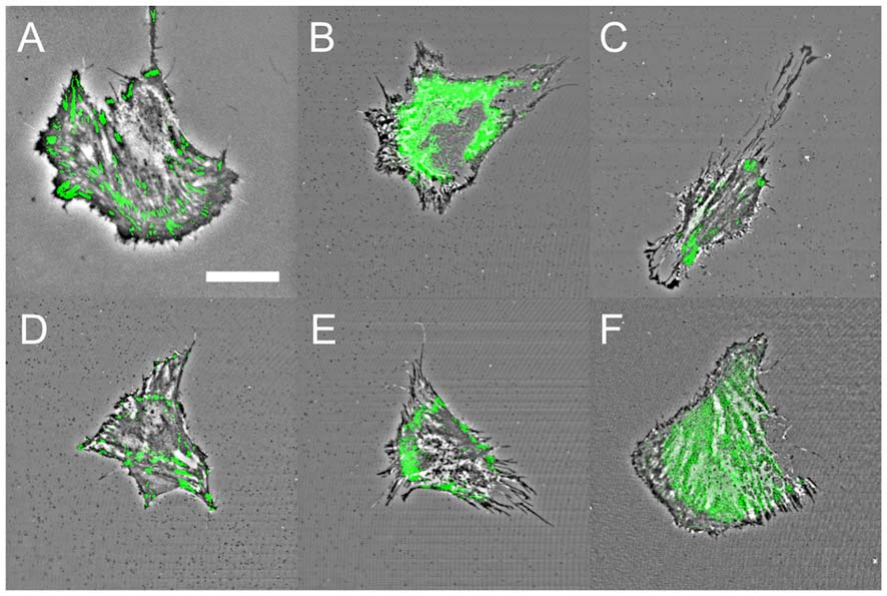
Overlay of IRM imaging and vinculin expression after 60 mins. Simultaneous IRM and fluorescence imaging of live cells expressing Vinculin-EGFP after exposure to inhibitors for 60 min. (**A**) Control (scale bar = 20 µm and applies to all), (**B**) Cyt-D, (**C**) nocodazole, (**D**) Y-27632A, (**E**) ML-7 and (**F**) blebbistatin treated cells. Cells treated with nocodazole, Y27632 and ML7 posses smaller but point-like FA structures, similar to the control. Vinculin becomes diffuse after treatment with CytD and blebbistatin.

**Figure 7:**
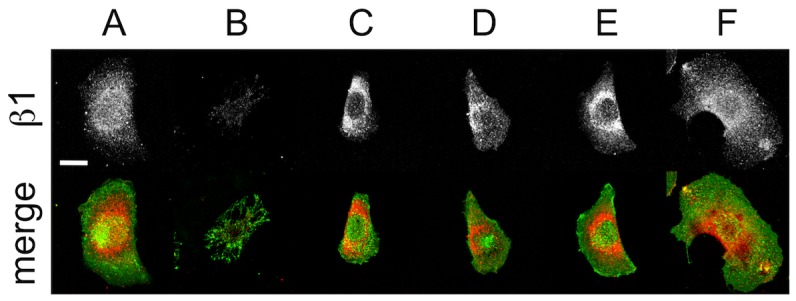
Fixed and stained images of ß1-integrin and the membrane of C2C12 cells. Cells were exposed to inhibitors for 60 min and then fixed and stained for the presence of β1-integrin (greyscale and red in the merge) and the plasma membrane (green in the merge). LSCM images were acquired at the cell-substrate interface. (**A**) Control (scale bar = 20 µm and applies to all), (**B**) Cyt-D, (**C**) nocodazole, (**D**) Y-27632A, (**E**) ML-7 and (**F**) blebbistatin treated cells. In all cases, integrin-ß1 is well distributed over the cell contact area. However, after 60 min of CytD treatment a significant decrease in integrin-ß1 was observed, correlating to a significant decrease in cell adhesion strength (Fig. 5).

**Figure 8:**
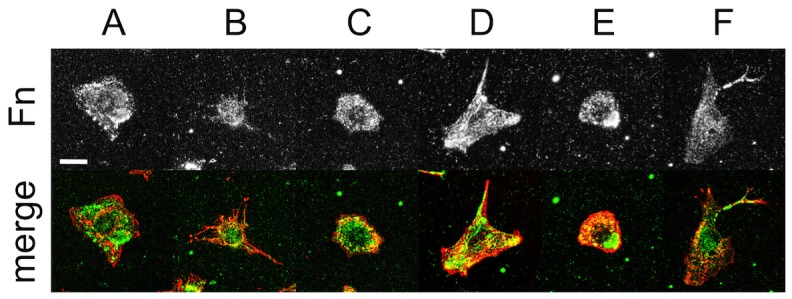
Fixed and stained images of fibronectin and the membrane of C2C12 cells. Cells were exposed to inhibitors for 60 min and then fixed and stained for the presence of fibronectin (Fn, greyscale and green in the merge) and the plasma membrane (red in the merge). LSCM images were acquired at the cell-substrate interface. (**A**) Control (scale bar = 20 µm and applies to all), (**B**) Cyt-D, (**C**) nocodazole, (**D**) Y-27632A, (**E**) ML-7 and (**F**) blebbistatin treated cells. In all cases, cell-secreted fibronectin was distributed over the substrate and found in higher concentrations underneath the cell body.

FAs, integrins and extracellular matrix proteins are critical structures that link the CSK to the extracellular environment. In order to probe the influence of the various inhibitors on the molecular basis of cellular CCA and adhesion we performed IRM imaging on live cells transiently expressing vinculin-EGFP (Fig. 6). In untreated cells, vinculin-EGFP displays typical punctate morphologies at FA sites (known to be linked to actin stress fibres) and corresponds well to darker regions of the IRM image as expected (Fig. 6A). However, there are a number of lower intensity regions in the IRM image with no vinculin-EGFP content. Interestingly, cells treated with Y27632 continue to display point-like vinculin-rich structures, though smaller in size and number (Fig. 6D). Nocodazole (Fig. 6C) and ML7 (Fig. 6E) treatment reduced the vinculin to only a few point-like structures whereas CytD (Fig. 6B) and blebbistatin (Fig. 6F) resulted in a diffuse distribution of vinculin-EGFP throughout the cell. As shown above (Fig. 3), treatment with the calcium chelator, EDTA, resulted in the complete detachment of cells from the substrate (under no flow). Therefore, cell-substrate adhesion is clearly dependent on the presence of calcium, implicating integrin function [3], [4]. We also fixed and methanol extracted cells to image the distribution of β1-integrin and cell-secreted fibronectin on the surface after 60 min of inhibitor treatment. In all cases LSCM images were collected at the planes containing the basal membrane and substrate in order to examine β1-integrin (Fig. 7) and fibronectin (Fig. 8) at the cell-substrate interface. After CytD treatment there is a complete loss of β1-integrin (Fig. 7B), but in all other cases it is found throughout the total cell contact area. β1-integrin was observed distributed diffusely throughout the cell spreading area, rather than localized in fibrillar or focal sites, as expected for C2C12 cells cultured on non-functionalized glass substrates for only 24 hours [43], [44]. After 24 hours of culture, cell-secreted fibronectin was found deposited on the glass substrate and in higher amounts beneath the cells before and after treatment with inhibitors. No fibronectin staining was observed on glass substrates without cells, which excludes the possibility of non-specific binding of the primary and secondary antibodies (data not shown). Taken-together, the results presented in this study reveal a complex relationship between the inhibition of various structures and pathways in the cell and changes in CCA, absolute cell size, cell adhesion and the presence and distribution of FA sites, β1-integrin and cell-secreted fibronectin. The results are summarized in Table 2 and are discussed further below.

**Table 2:** Changes in CCA, Cell Area, Adhesion, FAs and ß1-integrin for control cells and cells exposed to various inhibitors for 60 min (↓ = decrease, NC = no change from the control, D = diffuse, PL = point-like, NP = not present and P = present).

Treatment	CCA	Cell Area	Adhesion	FAs	ß1
Control	NC	NC	↓9%	PL	P
CytD	↓	↓	↓100%	D	NP
Nocodazole	↓	↓	↓48%	PL	P
Y27632	↓	↓	↓9%	PL	P
ML7	↓	↓	↓23%	PL	P
Blebbistatin	↓	NC	↓23%	D	P

## Discussion

Myoblasts are muscle precursor cells that can be mechanically stimulated to fuse and form myotubes and eventually new muscle tissue [32]–[36]. This process relies on the ability of these cells to sense mechanical properties and mechanical cues in their surrounding microenvironment [37]. This ability requires robust cell adhesion to the microenvironment via integrins and FA sites as well as an intact CSK for the transmission and conversion of mechanical information into biochemical signaling [2], [8], [9]. Importantly, when cells are exposed to physical stretch and compression this results in CSK remodeling, depolymerization and fluidization [32]–[36], [39], [40] which will change the nature of adhesion to the microenvironment. It remains unclear if myoblasts can maintain physical adhesion and contact to a substrate during CSK remodeling even though these processes are fundamentally linked to their function. Therefore, in this study we utilized IRM to examine the contribution of the CSK and acto-myosin contractility on the maintenance of cell-substrate contact and adhesion in C2C12 mouse myoblasts. We employed five inhibitors to examine the early response of myoblasts to actin and MT CSK remodeling as well as the inhibition of acto-myosin contractility regulators.

In previous studies, the relationship between total cell contact area and adhesion strength has been explored by confining cells to microprinted islands of adhesive molecules [14]–[16]. This allows the total contact area of the cell to be defined by the island size. In contrast, we employed IRM imaging to only determine the area occupied by the cell membrane close to the substrate, which we define as the close contact area (CCA). In this study, we examined the relationship of CCA with cell adhesion regardless of area defined by the cell membrane contour. This provides a more direct measure of what fraction of the cell remains in close physical contact with the substrate during dynamic remodeling of the cytoskeleton and if this contact corresponds to adhesion strength. Five specific inhibitors were employed in this study to remodel the actin CSK (CytD), the MT CSK (nocodazole) and inhibit regulators of acto-myosin contractility such as ROCK (Y27632), MLCK (ML7) and myoII (blebbistatin). Each caused distinct morphological changes in the cytoarchitecture. CytD causes a total loss of F-actin while leaving MTs intact. Nocodazole results in the collapse and depolymerization of MTs while leaving F-actin intact. Y27632 and ML7 both inhibit upstream regulators of myoII (ROCK and MLCK, respectively) and cause a loss of acto-myosin contractility. In addition we also inhibited myoII directly with blebbistatin. The loss of acto-myosin contractility results in the partial loss of a well-defined actin CSK while leaving MTs intact. Moreover, the loss of intact actin stress fibres in response to CytD and blebbistatin treatment results in the complete loss of intact FA sites. Other treatments only resulted in a decrease in the number and size of FA sites. Conversely, β1-integrin was found distributed throughout the total cell area (defined by the cell contour) after treatment with each inhibitor with the exception of CytD, in which there was a complete loss of β1-integrin.

However, although each inhibitor caused clear morphological changes in the cytoarchitecture and adhesion sites we only observed a ∼13% decrease in CCA with no statistically significant dependence on the inhibitor used. In each case, with the exception of blebbistatin, cells also decreased by ∼50% in total size relative to the control. Therefore, the ratio of CCA to absolute cell size increases during exposure to these inhibitors except for blebbistatin in which the ratio decreases. This implies that the proportion of the cell area in close contact with the surface actually increases in response to CSK, ROCK and MLCK inhibition but decreases in response to direct myoII inhibition. We also confirmed that the decrease in CCA was not due to cells contracting and moving away from the surface, as the average cell heights remain constant with each treatment. In order to examine if changes in CCA are reflected in adhesion strength, we subjected cells to a shear flow assay. Cells were first treated with each inhibitor for 60 mins prior to exposure to a shear stress. Although each inhibitor produced a similar decrease in cell CCA, cell adhesion had a clear dependence on the inhibitor used. Loss of the actin or microtubule CSK resulted in a 100% or ∼50% decrease in cell adhesion respectively. On the other hand inhibition of ROCK did not cause any significant change in cell adhesion. Whereas inhibition of MLCK or direct inhibition of myoII caused a ∼25% decrease in cell adhesion. This implies that though MLCK and ROCK both act to promote myoII contractility, it appears that MLCK may play a larger role in the maintenance of downstream cell adhesion. In each case, cell-secreted fibronectin was found distributed over the glass surface and in higher amounts directly underneath the cell body. Therefore, with the exception of CytD-treated cells, adhesion is likely maintained through integrin mediated links to underlying ECM proteins.

In brief, the CCA dynamics described above do not tend to correlate very well with adhesion strength, which has been previously shown to be dependent on the β1-integrin expression and intact vinculin-rich FAs [16]–[19]. As expected, we found that the strength of adhesion is correlated with the interplay between β1-integrin expression and the existence of intact vinculin-rich FAs. Indeed, depolymerization of actin CSK induced a loss of ß1-integrin expression along with point-like FAs. These findings suggest that the actin CSK is important for cell contact, cell-substrate adhesion strength and cell FA maturation. However, the MT CSK has also an equally important role in governing cell contact and adhesion, showing a significant reduction in cell-substrate adhesion strength in MT-depleted cells. Although the role of MTs in governing cell mechanics and cell adhesion are often ignored, an increasing number of studies have shown that they play a crucial role in governing cell traction forces and other mechanical dynamics [45]–[47] as they form part of the highly cross-linked CSK network in the cell. Interestingly, inhibition of myoII by blebbistatin also induced a significant decrease in cell adhesion but maintained a significantly stronger adhesion than actin-deprived cells despite the loss of point-like FAs. It has been shown that the recruitment of vinculin to FAs enhances adhesion in fibroblast cells [16]. Our findings demonstrate that intact and point-like FAs are not necessary for maintaining cell adhesion but β1-integrin is essential. Though each inhibitor used in this study had a clear effect on cell adhesion, it is evident that they do not disrupt the majority of cell contacts [24]. Clearly, FAs can be maintained in the absence of an intact MT CSK and during the inhibition of ROCK and MLCK [9], [48]. However, the dynamics of FAs during maturation, motility and actin contractility require the activity of myoII [1], [9], [48].

We have found that there is a complex relationship between cellular CCA, cell-adhesion and regulators of cytoskeletal remodeling and acto-myosin contractility. These structures are critical in myoblast cells that possess distinct mechanotransduction pathways, which are activated in response to local mechanical forces [37], [49], [50]. IRM imaging has revealed that the cellular CCA is decreased by ∼13% after 60 mins of exposure to relatively high concentrations of inhibitors. Regardless of the dramatic remodeling of the cytoarchitecture, myoblasts are still able to maintain a large degree of contact and adhesion to the substrate [24]. Laminin specific dystroglycan complexes and hemidesmosomes also play an important role in the adhesion and physiology of myoblasts and myotubes [37], [38]. The presence of specific integrin proteins in dystroglycan complexes and hemidesmosomes provides binding specificity to laminin, which is found in the ECM [51]. Importantly, myoblasts do not form dystroglycan complexes which are reserved for post-fusion myotubes [52]. In the case of hemidesmosomes, these protein complexes also form a link to laminin by mediating the interaction with intermediate filaments [53]. However, the myoblasts used in this study were cultured on unfunctionalized glass, which eliminates the contribution these complexes, in addition to the low expression of dystroglycan complexes in non-fused single myoblast cells [52]. Regardless, *in vivo*, these structures would only act to enhance the ability of myoblasts to maintain adhesion during physiologically or mechanically mediated CSK remodeling. Although the inhibitors used in this study represent extreme conditions, it is clear that myoblasts can still maintain close contact with the substrate. Extensive, future work can be undertaken to further elucidate a detailed mechanistic picture of which proteins are involved in the maintenance of CCA, FA sites and adhesion strength during cytoskeletal remodeling. However, our results clearly implicate the role of integrin-mediated links to the ECM. Emerging approaches might include assessing changes in FA and ECM composition, specifically at the cell-surface interface, by employing mass-spectroscopy and proteomic strategies [54], [55]. As recently shown, FA sites are extremely complex and can consist of hundreds of different proteins which may change in abundance in response to inhibitors and stress [54], [55]. This flexibility may permit the maintenance of FAs, CCA and adhesion during periods of stress.

The ability to maintain cell-substrate contact and adhesion under mechanical or biochemical stress may be part of a coping mechanism which enables myoblasts to quickly recover after removal of such stress [24]. In this study, we have shown that although the physiology of myoblast cells requires the integration of a mechanical and biochemical continuum between the ECM, integrins, FAs and the CSK, it is not necessarily required to maintain cell-substrate contact during short periods of stress. It appears that myoblasts are able to maintain a high degree of physical contact with the microenvironment during cytoarchitectural remodeling. This is important to myoblast function as mechanical stretch and compression causes large changes in CSK morphology and acto-myosin contractility [12], [56]. Therefore, although cell-substrate contact, adhesion and mechanosensitivity/mechanotransduction pathways are functionally integrated, they clearly posses distinct and time-dependent cytoarchitectural requirements.

## Materials and Methods

### Cell Culture and Transfections

C2C12 mouse myoblast cells were obtained from the American Type Culture Collection (CRL-1772). Cells were maintained in a 37°C, 5% CO_2_ incubator and cultured in DMEM with 10% fetal bovine serum (FBS) and 50 µg/ml streptomycin and 50 U/ml penicillin antibiotics (all from Hyclone Laboratories Inc.). For microscopy experiments cells were plated in 35 mm glass bottom dishes (Mat Tek) the day before experiments. In some cases C2C12 cells were transfected vinculin-GFP (kindly provided by Benny Geiger) using Lipofectamine 2000 (Invitrogen) according to the manufacturer’s instructions.

### Inhibitors

Cells were treated with either cytochalasin-D dissolved in dH_2_O (15 µM), nocodazole dissolved in DMSO (15 µM), Y27632 dissolved in dH_2_O (15 µM), ML-7 dissolved in dH_2_O (40 µM) or blebbistatin dissolved in DMSO (15 µM). All inhibitors were purchased from Sigma and the concentrations noted are the final concentrations in the cell culture dish.

### Immunofluorescence Staining

Briefly, cells were first fixed with 3.5% paraformaldehyde and permeabilized with Triton X-100 at 37°C. Staining for actin was accomplished with Phalloidin Alexa Fluor 546 (Invitrogen). Following this MTs were stained on ice with a mouse monoclonal anti-á-tubulin (Sigma) primary antibody and an Alexa Fluor 488 rabbit anti-mouse immunoglobin (Invitrogen) secondary antibody. DNA was labeled with DAPI (Invitrogen), also on ice. For staining of ß1-integrin or fibronectin cells were first fixed by parafromaldehyde and subsequently extracted by methanol. For integrin staining, anti-β1-integrin antibody [P5D2] (Abcam) and Alexa Fluor 546 rabbit anti-mouse immunoglobin (Invitrogen) were used as primary and secondary antibodies, respectively. Cells were also stained with wheat germ agglutinin coupled to Oregon Green 488 (Invitrogen) to reveal the cell membrane. For fibronectin staining, anti-human fibronectin antibody F3648 (Sigma) and Alexa Fluor 647 goat-anti-rabbit immunoglobulin (Invitrogen) were used, respectively. Cells were also stained with wheat germ agglutinin coupled to TRITC (Invitrogen) to reveal the cell boundaries.

### Microscopy

All images of living and fixed cells were acquired on a TiE A1-R laser scanning confocal microscope (Nikon) with a 60X/NA1.4 oil immersion lens. Images of fixed cells were acquired with a standard LSCM configuration with appropriate laser lines and filter blocks. IRM imaging was performed with a 640 nm diode laser in a standard LSCM IRM configuration [20], in which the excitation and emission light is of the same wavelength. IRM time-lapse imaging was performed with a perfect focus system (Nikon) to prevent focus drift. Cells were maintained at 37°C in a temperature-controlled stage and buffered in order to maintain pH. Images were acquired every 15 mins for a total of 60 mins.

### Image Analysis

All images were processed and analyzed with ImageJ (http://rsbweb.nih.gov/ij/). Brightness/contrast adjustments were the only processing steps applied to raw IRM, immunofluorescence or vinculin-EGFP images presented in this study. For quantification of IRM images, low intensity regions of the image were characterized to record changes in close cellular contact with the surface (described in the main text). To isolate these close contact regions and determine their total area we first created an image stack containing a particular cell imaged at 0, 15, 30, 45 and 60 min. We then applied a FFT band-pass filter followed by a background lightening in order to isolate the cell from the background. Thresholding was applied to create a binary image mask of the contact regions of the cell. Finally, any pixels corresponding to regions of the cell in close contact with the surface in the resultant binary image were summed to calculate the change in close contact area as a function of time.

### Parallel Plate Flow Assay

A polydimethylsiloxane (PDMS) microfluidic flow chamber, 1000 µm in width, 160 µm in height and 20 mm long, was produced using standard microfabrication techniques briefly described here. First a negative master was made with a SU-8 photoresist. Liquid-state 1∶10 ratio PDMS was poured on the master, solidified by baking at 80°C for at least 2 hours and then carefully peeled off. Inlets and outlets, 0.75 mm in diameter, were punched at both ends of the chamber. The PDMS layer was bonded to a standard glass microscope slide after being plasma treatment (100 W for 30 sec), creating an enclosed channel. Finally, tubing was attached to the inlets and outlets allowing media to be flowed through the chamber. Cells were cultured inside the chamber overnight and the next day cells were first exposed to each inhibitor for 60 min under no flow. The cells were exposed to an average flow of 330 µl/s, for 30 min, which corresponded to a shear stress at the wall of the chamber of 65 Dyne/cm^2^ [42]. All experiments were preformed inside a cell culture incubator. Using a microscope, cells were counted from the same region of interest before and after exposure to shear stress.

### Statistical Tests

All values quoted in this study are the average ± standard deviation. For comparisons between populations of data a student’s t-test and one-way ANOVA combined with a Tukey post-test were performed to determine statistical significance (á = 0.05).

## References

[1] Parsons JT, Horwitz AR, Schwartz MA (2010) Cell adhesion: integrating cytoskeletal dynamics and cellular tension. Nat Rev Mol Cell Biol 11: 633–643. 20729930 10.1038/nrm2957 PMC2992881

[2] Vicente-Manzanares M, Choi CK, Horwitz AR (2009) Integrins in cell migration–the actin connection. J Cell Sci 122: 199–206. 19118212 10.1242/jcs.018564 PMC2714416

[3] Takada Y, Ye X, Simon S (2007) The integrins. Genome Biol 8: 215. 17543136 10.1186/gb-2007-8-5-215 PMC1929136

[4] Arnaout MA, Goodman SL, Xiong JP (2007) Structure and mechanics of integrin-based cell adhesion. Curr Opin Cell Biol 19: 495–507. 17928215 10.1016/j.ceb.2007.08.002 PMC2443699

[5] Garcia-Alvarez B, de Pereda JM, Calderwood DA, Ulmer TS, Critchley D, et al (2003) Structural determinants of integrin recognition by talin. Mol Cell 11: 49–58. 12535520 10.1016/s1097-2765(02)00823-7

[6] Bakolitsa C, Cohen DM, Bankston LA, Bobkov AA, Cadwell GW, et al (2004) Structural basis for vinculin activation at sites of cell adhesion. Nature 430: 583–586. 15195105 10.1038/nature02610

[7] Giannone G, Jiang G, Sutton DH, Critchley DR, Sheetz MP (2003) Talin1 is critical for force-dependent reinforcement of initial integrin-cytoskeleton bonds but not tyrosine kinase activation. J Cell Biol 163: 409–419. 14581461 10.1083/jcb.200302001 PMC2173516

[8] Chen CS (2008) Mechanotransduction - a field pulling together? J Cell Sci 121: 3285–3292. 18843115 10.1242/jcs.023507

[9] Vicente-Manzanares M, Ma X, Adelstein RS, Horwitz AR (2009) Non-muscle myosin II takes centre stage in cell adhesion and migration. Nat Rev Mol Cell Biol 10: 778–790. 19851336 10.1038/nrm2786 PMC2834236

[10] Geiger B, Bershadsky A (2001) Assembly and mechanosensory function of focal contacts. Curr Opin Cell Biol 13: 584–592. 11544027 10.1016/s0955-0674(00)00255-6

[11] Riveline D, Zamir E, Balaban NQ, Schwarz US, Ishizaki T, et al (2001) Focal contacts as mechanosensors: externally applied local mechanical force induces growth of focal contacts by an mDia1-dependent and ROCK-independent mechanism. J Cell Biol 153: 1175–1186. 11402062 10.1083/jcb.153.6.1175 PMC2192034

[12] Humphries JD, Wang P, Streuli C, Geiger B, Humphries MJ, et al (2007) Vinculin controls focal adhesion formation by direct interactions with talin and actin. J Cell Biol 179: 1043–1057. 18056416 10.1083/jcb.200703036 PMC2099183

[13] Pasapera AM, Schneider IC, Rericha E, Schlaepfer DD, Waterman CM (2010) Myosin II activity regulates vinculin recruitment to focal adhesions through FAK-mediated paxillin phosphorylation. J Cell Biol 188: 877–890. 20308429 10.1083/jcb.200906012 PMC2845065

[14] Dumbauld DW, Shin H, Gallant ND, Michael KE, Radhakrishna H, et al (2010) Contractility modulates cell adhesion strengthening through focal adhesion kinase and assembly of vinculin-containing focal adhesions. J Cell Physiol 223: 746–756. 20205236 10.1002/jcp.22084 PMC2874193

[15] Dumbauld DW, Michael KE, Hanks SK, Garcia AJ (2010) Focal adhesion kinase-dependent regulation of adhesive forces involves vinculin recruitment to focal adhesions. Biol Cell 102: 203–213. 19883375 10.1042/BC20090104 PMC4915345

[16] Gallant ND, Michael KE, Garcia AJ (2005) Cell adhesion strengthening: contributions of adhesive area, integrin binding, and focal adhesion assembly. Mol Biol Cell 16: 4329–4340. 16000373 10.1091/mbc.E05-02-0170 PMC1196341

[17] Garcia AJ, Huber F, Boettiger D (1998) Force required to break alpha5beta1 integrin-fibronectin bonds in intact adherent cells is sensitive to integrin activation state. J Biol Chem 273: 10988–10993. 9556578 10.1074/jbc.273.18.10988

[18] Michael KE, Dumbauld DW, Burns KL, Hanks SK, Garcia AJ (2009) Focal adhesion kinase modulates cell adhesion strengthening via integrin activation. Mol Biol Cell 20: 2508–2519. 19297531 10.1091/mbc.E08-01-0076 PMC2675629

[19] Shi Q, Boettiger D (2003) A novel mode for integrin-mediated signaling: tethering is required for phosphorylation of FAK Y397. Mol Biol Cell 14: 4306–4315. 12960434 10.1091/mbc.E03-01-0046 PMC207021

[20] Barr VA, Bunnell SC (2009) Interference reflection microscopy. Curr Protoc Cell Biol Chapter 4: Unit 4 23. 10.1002/0471143030.cb0423s45 PMC2824538 20013754

[21] Verschueren H (1985) Interference reflection microscopy in cell biology: methodology and applications. J Cell Sci 75: 279–301. 3900106 10.1242/jcs.75.1.279

[22] Limozin L, Sengupta K (2009) Quantitative reflection interference contrast microscopy (RICM) in soft matter and cell adhesion. Chemphyschem 10: 2752–2768. 19816893 10.1002/cphc.200900601

[23] Holt MR, Calle Y, Sutton DH, Critchley DR, Jones GE, et al (2008) Quantifying cell-matrix adhesion dynamics in living cells using interference reflection microscopy. J Microsc 232: 73–81. 19017203 10.1111/j.1365-2818.2008.02069.x

[24] Choi CK, Margraves CH, English AE, Kihm KD (2008) Multicontrast microscopy technique to dynamically fingerprint live-cell focal contacts during exposure and replacement of a cytotoxic medium. J Biomed Opt 13: 054069. 19021447 10.1117/1.2993143

[25] Wechezak AR, Wight TN, Viggers RF, Sauvage LR (1989) Endothelial adherence under shear stress is dependent upon microfilament reorganization. J Cell Physiol 139: 136–146. 2708451 10.1002/jcp.1041390120

[26] Zand MS, Albrecht-Buehler G (1989) Long-term observation of cultured cells by interference-reflection microscopy: near-infrared illumination and Y-contrast image processing. Cell Motil Cytoskeleton 13: 94–103. 2766364 10.1002/cm.970130204

[27] Geiger B (1979) A 130 K protein from chicken gizzard: its localization at the termini of microfilament bundles in cultured chicken cells. Cell 18: 193–205. 574428 10.1016/0092-8674(79)90368-4

[28] Curtis AS (1964) The Mechanism of Adhesion of Cells to Glass. A Study by Interference Reflection Microscopy. J Cell Biol 20: 199–215. 14126869 10.1083/jcb.20.2.199 PMC2106393

[29] Ishiguro K, Kadomatsu K, Kojima T, Muramatsu H, Tsuzuki S, et al (2000) Syndecan-4 deficiency impairs focal adhesion formation only under restricted conditions. J Biol Chem 275: 5249–5252. 10681494 10.1074/jbc.275.8.5249

[30] Grinnell F (1986) Focal adhesion sites and the removal of substratum-bound fibronectin. J Cell Biol 103: 2697–2706. 2947902 10.1083/jcb.103.6.2697 PMC2114597

[31] Couchman JR, Yates J, King RJ, Badley RA (1981) Changes in microfilament and focal adhesion distribution with loss of androgen responsiveness in cultured mammary tumor cells. Cancer Res 41: 263–269. 7192599

[32] Collinsworth AM, Torgan CE, Nagda SN, Rajalingam RJ, Kraus WE, et al (2000) Orientation and length of mammalian skeletal myocytes in response to a unidirectional stretch. Cell and Tissue Research 302: 243–251. 11131135 10.1007/s004410000224

[33] Goldspink DF, Cox VM, Smith SK, Eaves LA, Osbaldeston NJ, et al (1995) Muscle Growth in Response to Mechanical Stimuli. American Journal of Physiology-Endocrinology and Metabolism 31: E288–E297. 10.1152/ajpendo.1995.268.2.E288 7532362

[34] Goldspink DF, Easton J, Winterburn SK, Williams PE, Goldspink GE (1991) The Role of Passive Stretch and Repetitive Electrical-Stimulation in Preventing Skeletal-Muscle Atrophy While Reprogramming Gene-Expression to Improve Fatigue Resistance. Journal of Cardiac Surgery 6: 218–224. 1807507 10.1111/jocs.1991.6.1s.218

[35] Vandenburgh HH, Hatfaludy S, Karlisch P, Shansky J (1989) Skeletal-Muscle Growth Is Stimulated by Intermittent Stretch-Relaxation in Tissue-Culture. American Journal of Physiology 256: C674–C682. 2923199 10.1152/ajpcell.1989.256.3.C674

[36] Vandenburgh HH, Swasdison S, Karlisch P (1991) Computer-Aided Mechanogenesis of Skeletal-Muscle Organs from Single Cells-Invitro. Faseb Journal 5: 2860–2867. 1916108 10.1096/fasebj.5.13.1916108

[37] Engler AJ, Griffin MA, Sen S, Bonnemann CG, Sweeney HL, et al (2004) Myotubes differentiate optimally on substrates with tissue-like stiffness: pathological implications for soft or stiff microenvironments. J Cell Biol 166: 877–887. 15364962 10.1083/jcb.200405004 PMC2172122

[38] Griffin MA, Sen S, Sweeney HL, Discher DE (2004) Adhesion-contractile balance in myocyte differentiation. J Cell Sci 117: 5855–5863. 15522893 10.1242/jcs.01496

[39] Krishnan R, Park CY, Lin YC, Mead J, Jaspers RT, et al (2009) Reinforcement versus fluidization in cytoskeletal mechanoresponsiveness. PLoS One 4: e5486. 19424501 10.1371/journal.pone.0005486 PMC2675060

[40] Chen C, Krishnan R, Zhou E, Ramachandran A, Tambe D, et al (2010) Fluidization and resolidification of the human bladder smooth muscle cell in response to transient stretch. PLoS One 5: e12035. 20700509 10.1371/journal.pone.0012035 PMC2917357

[41] Dhawan J, Helfman DM (2004) Modulation of acto-myosin contractility in skeletal muscle myoblasts uncouples growth arrest from differentiation. J Cell Sci 117: 3735–3748. 15252113 10.1242/jcs.01197

[42] Bacabac RG, Smit TH, Cowin SC, Van Loon JJWA, Nieuwstadt FTM, et al (2005) Dynamic shear stress in parallel-plate flow chambers. Journal of Biomechanics 38: 159–167. 15519352 10.1016/j.jbiomech.2004.03.020

[43] Neveux I, Doe J, Leblanc N, Valencik ML (2010) Influence of the extracellular matrix and integrins on volume-sensitive osmolyte anion channels in C2C12 myoblasts. Am J Physiol Cell Physiol 298: C1006–1017. 20164377 10.1152/ajpcell.00359.2009 PMC2867394

[44] Borm B, Requardt RP, Herzog V, Kirfel G (2005) Membrane ruffles in cell migration: indicators of inefficient lamellipodia adhesion and compartments of actin filament reorganization. Experimental cell research 302: 83–95. 15541728 10.1016/j.yexcr.2004.08.034

[45] Kaverina I, Krylyshkina O, Small JV (1999) Microtubule targeting of substrate contacts promotes their relaxation and dissociation. J Cell Biol 146: 1033–1044. 10477757 10.1083/jcb.146.5.1033 PMC2169483

[46] Even-Ram S, Doyle AD, Conti MA, Matsumoto K, Adelstein RS, et al (2007) Myosin IIA regulates cell motility and actomyosin-microtubule crosstalk. Nat Cell Biol 9: 299–309. 17310241 10.1038/ncb1540

[47] Pelling AE, Veraitch FS, Chu CP, Mason C, Horton MA (2009) Mechanical dynamics of single cells during early apoptosis. Cell Motil Cytoskeleton 66: 409–422. 19492400 10.1002/cm.20391

[48] Choi CK, Vicente-Manzanares M, Zareno J, Whitmore LA, Mogilner A, et al (2008) Actin and alpha-actinin orchestrate the assembly and maturation of nascent adhesions in a myosin II motor-independent manner. Nat Cell Biol 10: 1039–1050. 19160484 10.1038/ncb1763 PMC2827253

[49] Sbrana F, Sassoli C, Meacci E, Nosi D, Squecco R, et al (2008) Role for stress fiber contraction in surface tension development and stretch-activated channel regulation in C2C12 myoblasts. Am J Physiol Cell Physiol 295: C160–172. 18480300 10.1152/ajpcell.00014.2008

[50] Formigli L, Meacci E, Sassoli C, Chellini F, Giannini R, et al (2005) Sphingosine 1-phosphate induces cytoskeletal reorganization in C2C12 myoblasts: physiological relevance for stress fibres in the modulation of ion current through stretch-activated channels. J Cell Sci 118: 1161–1171. 15728255 10.1242/jcs.01695

[51] Tsuruta D, Hopkinson SB, Jones JC (2003) Hemidesmosome protein dynamics in live epithelial cells. Cell Motil Cytoskeleton 54: 122–134. 12529858 10.1002/cm.10089

[52] Campbell KP (1995) Three muscular dystrophies: loss of cytoskeleton-extracellular matrix linkage. Cell 80: 675–679. 7889563 10.1016/0092-8674(95)90344-5

[53] Rezniczek GA, de Pereda JM, Reipert S, Wiche G (1998) Linking integrin alpha6beta4-based cell adhesion to the intermediate filament cytoskeleton: direct interaction between the beta4 subunit and plectin at multiple molecular sites. J Cell Biol 141: 209–225. 9531560 10.1083/jcb.141.1.209 PMC2132717

[54] Kuo JC, Han X, Hsiao CT, Yates JR, 3rd, Waterman CM (2011) Analysis of the myosin-II-responsive focal adhesion proteome reveals a role for beta-Pix in negative regulation of focal adhesion maturation. Nat Cell Biol 13: 383–393. 21423176 10.1038/ncb2216 PMC3279191

[55] Kuo JC, Han X, Yates JR, 3rd, Waterman CM (2012) Isolation of focal adhesion proteins for biochemical and proteomic analysis. Methods Mol Biol 757: 297–323. 21909920 10.1007/978-1-61779-166-6_19 PMC4158431

[56] Soltow QA, Lira VA, Betters JL, Long JH, Sellman JE, et al (2010) Nitric oxide regulates stretch-induced proliferation in C2C12 myoblasts. J Muscle Res Cell Motil 31: 215–225. 20717711 10.1007/s10974-010-9227-4

