# Correction: The Physical Interaction of Myoblasts with the Microenvironment during Remodeling of the Cytoarchitecture

**DOI:** 10.1371/annotation/4a6a7d2c-e898-481c-b1e1-f6f1ad7fc6e2

**Published:** 2013-05-06

**Authors:** Daniel J. Modulevsky, Dominique Tremblay, Corinne Gullekson, Nickolay V. Bukoreshtliev, Andrew E. Pelling

There was an error in the name of the fourth author.

The correct author name is: Nickolay V. Bukoreshtliev

The correct citation is: Modulevsky DJ, Tremblay D, Gullekson C, Bukoreshtliev NV, Pelling AE (2012) The Physical Interaction of Myoblasts with the Microenvironment during Remodeling of the Cytoarchitecture. PLoS ONE 7(9): e45329. doi:10.1371/journal.pone.0045329 

